# Patient characteristics and overall survival in patients with post-docetaxel metastatic castration-resistant prostate cancer in the community setting

**DOI:** 10.1007/s12032-017-1014-2

**Published:** 2017-08-10

**Authors:** William K. Oh, Raymond Miao, Francis Vekeman, Jennifer Sung, Wendy Y. Cheng, Marjolaine Gauthier-Loiselle, Ravinder Dhawan, Mei Sheng Duh

**Affiliations:** 1 Division of Hematology/Medical Oncology, The Tisch Cancer Institute, Icahn School of Medicine at Mount Sinai, New York, NY USA; 2 Sanofi US, Bridgewater, NJ USA; 3 Groupe d’analyse, Ltée, Montréal, QC Canada; 4 Analysis Group, Inc., Boston, MA USA

**Keywords:** mCRPC, Chemotherapy, AR-targeted therapy, Real-world, Cabazitaxel

## Abstract

It is unclear how treatment sequencing for metastatic castration-resistant prostate cancer (mCRPC) affects real-world patient outcomes. We assessed treatment sequences, patient characteristics and overall survival (OS) in post-docetaxel mCRPC patients. mCRPC patients receiving second-line cabazitaxel or androgen receptor-targeted therapy (ART; abiraterone/enzalutamide) post-docetaxel were identified using electronic medical records. OS was assessed from second-line therapy initiation using Cox regressions adjusting for: metastases; prostate-specific antigen (PSA); hemoglobin; alkaline phosphatase (ALP); albumin; second-line therapy initiation year. Following docetaxel (*n* = 629), 123 (19.6%) and 506 (80.4%) patients received cabazitaxel and ART, respectively. One hundred and ninety-five patients received additional treatments thereafter (54 following cabazitaxel; 141 following ART). Although patients receiving second-line cabazitaxel versus ART had similar disease characteristics at first-line therapy initiation, at second-line therapy initiation they had higher mean PSA (386.6 vs. 233.9 ng/mL) and ALP (182.0 vs. 167.3 u/L), lower mean hemoglobin (10.8 vs. 11.5 g/dL), and more frequently had intermediate/high-risk Halabi scores (61.8 vs. 48.4%); all *p* < 0.05. Overall, crude survival was not significantly different. Among Halabi high-risk patients, adjusted median OS was significantly longer in patients receiving cabazitaxel versus ART (HR 0.48; 95% CI 0.24–0.93; *p* = 0.030). Low albumin and hemoglobin led to similar findings (HR 0.43; 95% CI 0.23–0.80; *p* = 0.0077; HR 0.60; 95% CI 0.40–0.90; *p* = 0.014). Most post-docetaxel patients received second-line ART. Patients receiving second-line cabazitaxel had more high-risk features; however, second-line cabazitaxel administered after docetaxel may improve OS in patients with Halabi high-risk scores or low albumin/hemoglobin.

## Introduction

It has been estimated that 180,890 new cases of prostate cancer will be diagnosed in the USA in 2016, and that these will represent 21% of all new cancer diagnoses in American men [1]. Despite significant progress in the discovery of novel therapeutic agents, patients with metastatic castration-resistant prostate cancer (mCRPC) continue to have a poor prognosis, and their treatment remains a significant clinical challenge [2]. Until recently, treatment options that improve survival were limited to docetaxel-based regimens. However, recent advances have led to the approval of several new agents shown to improve survival, including the taxane chemotherapy, cabazitaxel, and the androgen receptor (AR)-targeted agents, abiraterone and enzalutamide [3]. As these three agents were developed simultaneously and largely in isolation, limited data are available on their optimal sequencing in the treatment of mCRPC.

A number of retrospective studies have sought to define the association between different treatment sequences of abiraterone, enzalutamide, docetaxel and cabazitaxel and patient outcomes such as overall survival (OS), progression-free survival (PFS) and prostate-specific antigen (PSA) response [4–8]. However, in the absence of prospective sequencing studies, the available literature assessing the optimal sequencing of treatments to achieve the best possible patient outcomes in mCRPC remains limited and inconclusive (as extensively reviewed by Lorente et al. [2]). For example, some studies have suggested that, in patients with mCRPC who have progressed on docetaxel, the sequence of subsequent therapies can affect patient outcomes. It has been shown that, after docetaxel, administration of cabazitaxel before, rather than after, AR-targeted therapy is associated with prolonged OS [9–11]. However, other studies have shown that cabazitaxel retains activity in patients who have received prior docetaxel and AR-targeted therapy suggesting that prior AR-targeted therapy does not influence the efficacy of cabazitaxel [12, 13].

Understanding the potential link between treatment sequences and patient outcomes is important to help achieve the most optimal patient benefit, particularly in the post-docetaxel setting; most patients with mCRPC have received docetaxel as part of their long-term treatment regimen as it is a standard treatment for patients with symptomatic metastases [14, 15]. In addition, recent studies have shown that a combination of androgen-deprivation therapy and docetaxel as first-line therapy for hormone-sensitive metastatic prostate cancer can improve OS, suggesting that in the future more patients may have received docetaxel by the time they have developed castration-resistant disease [16, 17]. In patients with mCRPC who have progressed after first-line docetaxel, it is unclear whether they would benefit from receiving a further line of chemotherapy (cabazitaxel) or second-line AR-targeted therapy. This study aimed to evaluate the real-world effectiveness of mCRPC treatment sequences. Specifically, we assessed whether the choice of second-line treatment post-docetaxel is associated with OS in patients with mCRPC in order to provide information on the optimal placement of cabazitaxel in the treatment sequence for patients with mCRPC.

## Methods

### Data source

This study used de-identified electronic medical records (EMR) data obtained from Altos Solutions, Inc (Silicon Valley, CA, USA). This database includes information from patients treated in community oncology practices that use OncoEMR™, an oncology-specific EMR system developed by Altos. The network of Altos includes 150 medical oncology practices across the USA and represents approximately 20% of all oncologists practicing in the community setting. Because our study involved retrospective analysis of existing data with no patient intervention or interaction, and patient data were de-identified, the New England Independent Review Board (NEIRB) determined that this study was exempt from NEIRB review (per Title 45 of CFR, Part 46).

### Study design

**Fig. 1:**
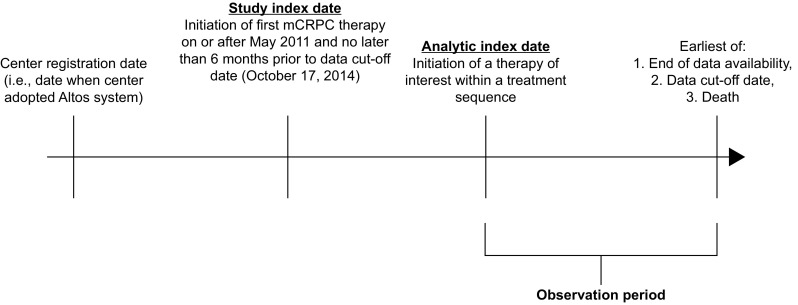
Study design. *mCRPC* metastatic castration-resistant prostate cancer

This study used a retrospective, longitudinal, open cohort design. The study index date was defined as the initiation of first-line docetaxel and was restricted to be no later than 6 months prior to the data cut-off date (October 17, 2014). To avoid selection bias based on treatment availability, the initiation of docetaxel was required to have occurred on or after May 2011 (when abiraterone was approved for use post-docetaxel in the USA). The analytic index date was defined as the initiation of second-line therapy, and the observation period began with the analytic index date and extended to the end of data availability (loss to follow-up), the data cut-off date or death, whichever came first (Fig. 1).

### Study sample

The study sample cohorts were driven by the observed treatment patterns among patients who received first-line docetaxel. Firstly, the types of therapies and treatment sequences received were characterized. Secondly, based on the observed sequence distribution, patients were stratified into two cohorts: (1) cabazitaxel cohort: patients who received first-line docetaxel, followed by second-line cabazitaxel and any or no subsequent treatment (2) AR-targeted therapy cohort: patients who received first-line docetaxel, followed by second-line AR-targeted therapy (abiraterone or enzalutamide) and any or no subsequent treatment. Patients who received a combination of AR-targeted therapy and chemotherapy in either the first- or second-line setting were excluded from the analysis.

### Eligibility criteria

Patients included in the study had at least one medical record with a diagnosis of prostate cancer and had received at least two mCRPC treatments of interest (docetaxel, cabazitaxel, abiraterone or enzalutamide) including docetaxel as first-line therapy, followed by any other treatment of interest in the second-line setting. Patients had to be at least 18 years old at the time of prostate cancer diagnosis.

### Study endpoints

Treatment effectiveness was assessed in terms of OS, which was defined as the time from initiation of second-line therapy to death from any cause.

### Statistical methods

Descriptive analyses were conducted to characterize the landscape of therapies that mCRPC patients received following first-line docetaxel. Descriptive statistics were generated to summarize patient demographic and clinical characteristics at the initiation of first- and second-line therapy. Comparisons between cohorts were conducted using Chi-squared tests for categorical variables and Wilcoxon rank-sum tests for continuous variables. OS was assessed using the Kaplan–Meier method and compared using log-rank tests. Multivariate Cox proportional hazard models were also conducted to compare OS between cohorts, adjusting for the year of the initiation of second-line therapy, site of metastasis, and PSA, hemoglobin, alkaline phosphatase (ALP) and albumin levels. OS was also assessed for specific patient subgroups based on Halabi risk score [18] at the initiation of second-line therapy, as well as its underlying prognostic factors including PSA (>50 ng/mL), hemoglobin (<11 g/dL), ALP (>1 × upper limit of normal [ULN]), lactate dehydrogenase (LDH; >1 × ULN), and albumin levels (<1 × lower limit of normal), use of opioids, and the presence of bone or visceral metastases. Statistical analyses were performed using SAS software version 9.3 or more recent.

## Results

### Study population and cohorts

**Fig. 2:**
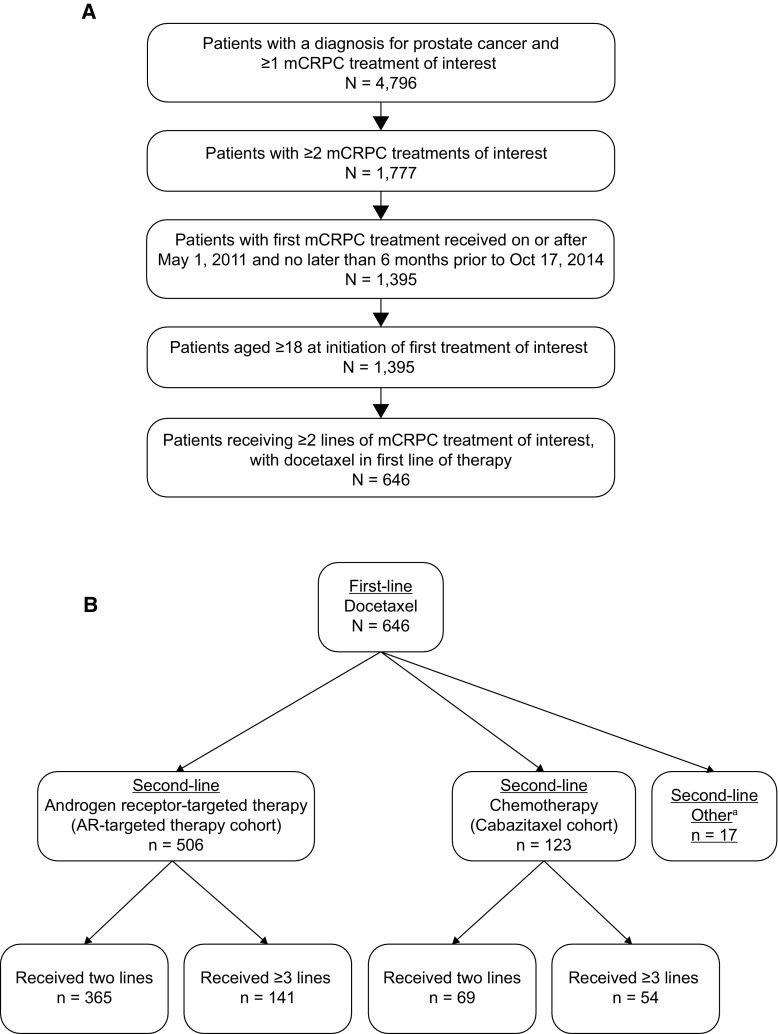
Study sample selection (**a**) and cohort allocation (**b**). *AR* andrgoen receptor, *mCRPC* metastatic castration-resistant prostate cancer. ^a^Excluded from the further analyses

A total of 646 patients received first-line docetaxel of which 629 received either cabazitaxel or AR-targeted therapy in the second-line setting (Fig. 2a, b). The remaining 17 patients received a combination of cabazitaxel and AR-targeted therapy as second-line treatment and were excluded from further analyses. Of the 629 eligible patients, 19.6% (*n* = 123) received cabazitaxel followed by any or no further treatment and 80.4% (*n* = 506) received AR-targeted therapy followed by any or no further treatment. Of the patients in the cabazitaxel cohort, 56.1% (*n*/*N* = 69/123) received no further therapy of interest following second-line cabazitaxel, whereas 72.1% of patients (*n*/*N* = 365/506) in the AR-targeted therapy cohort received no further treatment of interest following second-line AR-targeted therapy (Table 1). Of the patients in the AR-targeted therapy cohort receiving only two lines of therapy of interest (*n* = 365), 233 patients (63.8%) received second-line abiraterone, 129 (35.3%) received second-line enzalutamide and 3 (0.8%) received both AR-targeted agents in the second-line setting (Table 1). Among the 629 eligible patients who received second-line cabazitaxel or AR-targeted therapy, 434 patients (69.0%) received a total of two treatment lines of interest only, and 195 (31.0%) received a total of three or more treatment lines of interest (54 patients in the cabazitaxel cohort and 141 patients in the AR-targeted therapy cohort) (Fig. 2b).

**Table 1:** Treatment sequences received by patients with docetaxel as first observed mCRPC therapy

	*n* (%)
*Sequence of two therapies*	
Docetaxel → AR-targeted therapy	365 (81.8)
Abiraterone	233 (52.2)
Enzalutamide	129 (28.9)
Abiraterone/enzalutamide	3 (0.7)
Docetaxel → cabazitaxel	69 (15.5)
Other (e.g., cabazitaxel/AR-targeted therapy)	12 (2.7)
Total patients with a sequence of two therapies	446 (69.0)
*Sequence of three therapies*	
Docetaxel → AR-targeted therapy → alternative AR-targeted therapy	57 (37.5)
Abiraterone → enzalutamide	40 (26.3)
Enzalutamide → abiraterone	15 (9.9)
Abiraterone → abiraterone/enzalutamide	2 (1.3)
Docetaxel → AR-targeted therapy → chemotherapy	44 (28.9)
Abiraterone → cabazitaxel	20 (13.2)
Enzalutamide → cabazitaxel	13 (8.6)
Abiraterone → docetaxel	6 (3.9)
Enzalutamide → docetaxel	5 (3.3)
Docetaxel → AR-targeted therapy → other (e.g., chemotherapy/alternative AR-targeted therapy)	3 (2.0)
Docetaxel → cabazitaxel → AR-targeted therapy	42 (27.6)
Cabazitaxel → abiraterone	28 (18.4)
Cabazitaxel → enzalutamide	14 (9.2)
Docetaxel → cabazitaxel → other (e.g., chemotherapy/AR-targeted therapy)	1 (0.7)
Other (e.g., chemotherapy/AR-targeted therapy)	5 (3.3)
Total patients with a sequence of three therapies	152 (23.5)
*Sequence of four or more therapies*	
Total patients with a sequence of four therapies	37 (5.7)
Total patients with a sequence of five therapies	8 (1.2)
Total patients with a sequence of six therapies	3 (0.5)
Total	646 (100)
Patients with second-line cabazitaxel or AR-targeted therapy	629 (97.4)

*AR* androgen receptor, *mCRPC* metastatic castration-resistant prostate cancer

The median duration of first-line docetaxel therapy was similar between the two patient cohorts (4.2 months for both the cabazitaxel and AR-targeted therapy cohorts; *p* = 0.548) (Table 2). The median duration of second-line therapy was significantly shorter for those receiving second-line cabazitaxel versus second-line AR-targeted therapy (2.8 vs. 4.7 months; *p* = 0.001) (Table 3).

**Table 2:** Characteristics of patients receiving second-line cabazitaxel versus second-line androgen receptor-targeted therapy at initiation of first-line docetaxel therapy

Characteristics	Second-line therapy received		*p* value
	Cabazitaxel *N* = 123	AR-targeted therapy *N* = 506	
Age, years, mean ± SD (median)	70.8 ± 9.3 (72.0)	71.6 ± 9.0 (72.0)	0.503
Age, *n* (%)			
<65 years	29 (23.6)	109 (21.5)	0.625
65–74 years	46 (37.4)	190 (37.5)	0.975
≥75 years	48 (39.0)	207 (40.9)	0.703
ECOG performance status, *n* (%)			
0	24 (19.5)	74 (14.6)	0.180
1	11 (8.9)	62 (12.3)	0.304
2	2 (1.6)	17 (3.4)	0.314
3	1 (0.8)	2 (0.4)	0.546
Unknown	85 (69.1)	351 (69.4)	0.955
Number of metastases, mean ± SD (median)	1.1 ± 0.3 (1.0)	1.1 ± 0.4 (1.0)	0.505
Bone metastasis, *n* (%)	82 (66.7)	296 (58.5)	0.097
Visceral metastasis, *n* (%)	7 (5.7)	20 (4.0)	0.394
Lymph node metastasis, *n* (%)	3 (2.4)	29 (5.7)	0.136
Unknown, *n* (%)	38 (30.9)	193 (38.1)	0.135
PSA, ng/mL, mean ± SD (median)	359.7 ± 1211.2 (85.3)	262.3 ± 962.8 (63.4)	0.193
ALP, u/L, mean ± SD (median)	231.1 ± 233.9 (139.0)	206.4 ± 269.6 (111.0)	0.039*
LDH, u/L, mean ± SD (median)	373.8 ± 248.0 (256.0)	333.2 ± 448.1 (222.0)	0.048*
Albumin, g/dL, mean ± SD (median)	3.8 ± 0.4 (3.9)	3.9 ± 0.5 (3.9)	0.219
Hemoglobin, g/dL, mean ± SD (median)	11.7 ± 1.5 (11.9)	11.7 ± 1.6 (11.7)	0.999
Halabi risk score, mean ± SD (median)^a^	127.6 ± 51.8 (126.7)	126.1 ± 50.4 (121.6)	0.243
Low risk, *n* (%)	70 (56.9)	325 (64.2)	0.132
Intermediate risk, *n* (%)	43 (35.0)	139 (27.5)	0.100
High risk, *n* (%)	10 (8.1)	42 (8.3)	0.951
Opioid use in prior 365 days, *n* (%)	39 (31.7)	136 (26.9)	0.284
Duration of first-line therapy, months, mean ± SD (median)	5.4 ± 4.4 (4.2)	5.2 ± 4.4 (4.2)	0.548

*AR* androgen receptor, *ALP* alkaline phosphatase, *ECOG* Eastern Cooperative Oncology Group, *LDH* lactate dehydrogenase, *PSA* prostate-specific antigen, *SD* standard deviation

^a^Based on Halabi et al. [18]

* Denotes significant *p* value at the 5% level

**Table 3:** Characteristics of patients receiving second-line cabazitaxel versus second-line androgen receptor-targeted therapy at initiation of second-line therapy

Characteristics	Second-line therapy received		*p* value
	Cabazitaxel *N* = 123	AR-targeted therapy *N* = 506	
Age, years, mean ± SD (median)	71.5 ± 9.2 (72.0)	72.3 ± 9.0 (73.0)	0.478
Age, *n* (%)			
<65 years	27 (22.0)	98 (19.4)	0.520
65–74 years	42 (34.1)	188 (37.2)	0.534
≥75 years	54 (43.9)	220 (43.5)	0.932
ECOG performance status, *n* (%)			
0	15 (12.2)	60 (11.9)	0.918
1	19 (15.4)	83 (16.4)	0.796
2	7 (5.7)	24 (4.7)	0.663
3	0 (0.0)	15 (3.0)	–
Unknown	81 (65.9)	323 (63.8)	0.675
Number of metastases, mean ± SD (median)	1.1 ± 0.4 (1.0)	1.1 ± 0.4 (1.0)	0.860
Bone metastasis, *n* (%)	90 (73.2)	325 (64.2)	0.061
Visceral metastasis, *n* (%)	10 (8.1)	24 (4.7)	0.136
Lymph node metastasis, *n* (%)	3 (2.4)	35 (6.9)	0.062
Unknown, *n* (%)	30 (24.4)	160 (31.6)	0.117
PSA, ng/mL, mean ± SD (median)	386.6 ± 760.5 (126.6)	233.9 ± 659.4 (47.0)	0.001*
ALP, u/L, mean ± SD (median)	182.0 ± 189.7 (131.0)	167.3 ± 240.2 (91.0)	0.016*
LDH, u/L, mean ± SD (median)	347.7 ± 291.4 (241.0)	589.8 ± 1904.2 (221.0)	0.122
Albumin, g/dL, mean ± SD (median)	3.9 ± 3.4 (3.7)	3.9 ± 3.5 (3.8)	0.522
Hemoglobin, g/dL, mean ± SD (median)	10.8 ± 1.5 (10.9)	11.5 ± 2.0 (11.4)	<0.001*
Halabi risk score, mean ± SD (median)^a^	153.6 ± 49.2 (154.5)	143.8 ± 53.8 (136.4)	0.013*
Low risk, *n* (%)	47 (38.2)	261 (51.6)	0.008*
Intermediate risk, *n* (%)	55 (44.7)	169 (33.4)	0.019*
High risk, *n* (%)	21 (17.1)	76 (15.0)	0.572
Opioid use in prior 365 days, *n* (%)	53 (43.1)	231 (45.7)	0.609
Duration of second-line therapy, months, mean ± SD (median)	4.2 ± 1.9 (2.8)	6.1 ± 1.9 (4.7)	0.001*

*AR* androgen receptor, *ALP* alkaline phosphatase, *ECOG* Eastern Cooperative Oncology Group, *LDH* lactate dehydrogenase, *PSA* prostate-specific antigen, *SD* standard deviation

^a^Based on Halabi et al. [18]

* Denotes significant *p* value at the 5% level

### Patient baseline characteristics

At the initiation of first-line docetaxel therapy, patient characteristics were generally similar across cohorts. Only ALP and LDH were significantly different, with patients receiving second-line cabazitaxel presenting with higher mean levels versus patients receiving second-line AR-targeted therapy (ALP: 231.1 vs. 206.4 u/L, *p* = 0.039; LDH: 373.8 vs. 333.2 u/L, *p* = 0.048). This, however, did not translate into a significant difference in the mean Halabi risk score at baseline (127.6 vs. 126.1 points, *p* = 0.243, for the cabazitaxel and AR-targeted therapy cohorts, respectively) (Table 2).

At the initiation of second-line therapy, patients in the cabazitaxel cohort presented with poorer prognostic features compared with patients in the AR-targeted therapy cohort (Table 3). Of note, patients in the cabazitaxel cohort had significantly higher mean PSA and ALP levels (PSA: 386.6 vs. 233.9 ng/mL, *p* = 0.001; ALP: 182.0 vs. 167.3 u/L, *p* = 0.016), and significantly lower mean hemoglobin levels (10.8 vs. 11.5 g/dL, *p* < 0.001). The mean Halabi risk score was 153.6 versus 143.8 for the cabazitaxel and AR-targeted therapy cohorts, respectively (*p* = 0.013), and a greater proportion of patients in the cabazitaxel cohort was categorized into the intermediate- or high-risk Halabi groups (61.8 vs. 48.4%, *p* = 0.008).

### Overall survival

**Fig. 3:**
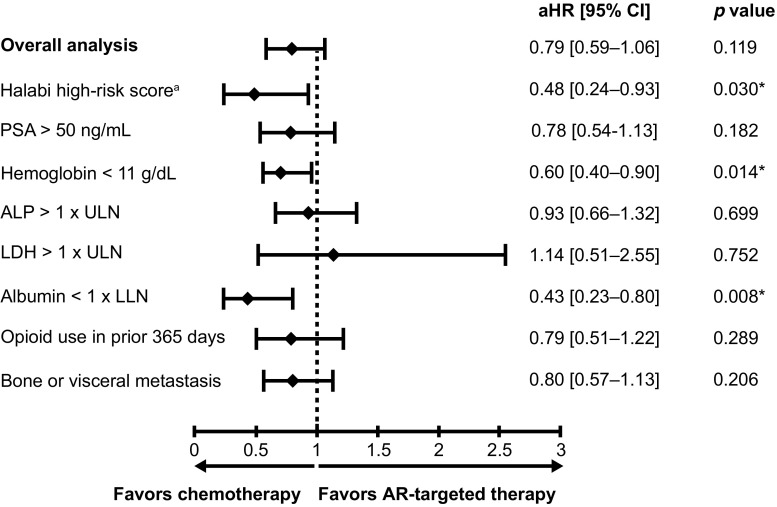
Adjusted hazard ratios for overall survival by subgroup for patients receiving second-line chemotherapy versus second-line androgen receptor-targeted therapy. *aHR* adjusted hazard ratio, *ALP* alkaline phosphatase, *CI* confidence interval, *LDH* lactate dehydrogenase, *LLN* lower limit of normal, *PSA* prostate-specific antigen, *ULN* upper limit of normal. ^a^ Based on Halabi et al. [18]. * Denotes significant *p* value at the 5% level

The difference in overall median OS for patients receiving second-line cabazitaxel versus patients receiving second-line AR-targeted therapy did not reach statistical significance (10.2 vs. 15.1 months; *p* = 0.155). Results from the multivariate Cox regression showed a trend for OS in favor of cabazitaxel, although the results did not reach statistical significance (adjusted hazard ratio for cabazitaxel vs. AR-targeted therapy: 0.79; 95% Confidence Interval [CI] 0.59–1.06, *p* = 0.119) (Fig. 3).

In the subgroup of patients defined as Halabi high risk, median OS was 6.9 months for patients receiving second-line cabazitaxel and 4.7 months for patients receiving second-line AR-targeted therapy (*p* = 0.320). After adjustment for observed differences in risk factors, median OS was significantly longer for Halabi high-risk patients receiving second-line cabazitaxel compared with Halabi high-risk patients receiving second-line AR-targeted therapy (Hazard Ratio [HR]: 0.48; 95% CI 0.24–0.93; *p* = 0.030). These findings, indicating a benefit with second-line cabazitaxel, were similar in the subgroups of patients with albumin levels below the lower limit of normal (HR: 0.43; 95% CI 0.23–0.80; *p* = 0.008) and hemoglobin levels <11 g/dL (HR: 0.60; 95% CI 0.40–0.90; *p* = 0.014) (Fig. 3).

## Discussion

This retrospective study described the real-world treatment sequences received by patients with mCRPC following first-line docetaxel and outlined the characteristics of patients who received either cabazitaxel or AR-targeted therapy as second-line treatment. This study also assessed the real-world effectiveness in terms of OS of cabazitaxel versus AR-targeted therapy in the post-docetaxel setting. Data showed that after first-line docetaxel, more patients received AR-targeted therapy as a second-line treatment versus cabazitaxel in the community oncology setting. The patients who received second-line cabazitaxel, post-docetaxel, had more high-risk features and a worse prognosis compared with the patients who received second-line AR-targeted therapy. This suggested that more patients with aggressive cancer progression were included in the cabazitaxel cohort, and that oncologists favored second-line chemotherapy as a treatment for these patients. In addition, our findings suggest that, for patients presenting with worse prognostic features, including a Halabi high-risk score, and low albumin and/or low hemoglobin levels, there may be an OS benefit associated with second-line cabazitaxel compared with second-line AR-targeted therapy, following first-line docetaxel. Trends favoring patients receiving second-line cabazitaxel were also observed in patients with PSA > 50 mg/mL and bone or visceral metastases at the initiation of second-line therapy, and in patients who had used opioid pain medication in the 365 days prior to second-line therapy; however, these trends were not significant.

Consistent with our findings, a number of recent studies have suggested that, in the post-docetaxel setting, there may be a survival benefit when receiving cabazitaxel earlier in the treatment regimen. One retrospective study, for example, found that among patients receiving three lines of therapy including first-line docetaxel, followed by second-line and third-line cabazitaxel or abiraterone, those receiving second-line cabazitaxel followed by third-line abiraterone experienced significantly prolonged OS compared with patients receiving the opposite sequence of second-line abiraterone followed by third-line cabazitaxel (median OS: 18.2 vs. 11.8 months; *p* = 0.021) [10]. Another study showed a survival benefit associated with receiving cabazitaxel prior to either abiraterone or enzalutamide in the post-docetaxel setting with a median OS of 49.0 months reported in patients receiving cabazitaxel prior to abiraterone or enzalutamide versus 20.8 months in patients receiving abiraterone or enzalutamide prior to cabazitaxel [9]. When other patient outcome parameters were assessed, such as PFS, among patients who received three lines of therapy, including first-line docetaxel, there was no difference in PFS for patients receiving second-line cabazitaxel followed by third-line abiraterone (4.6 months) versus second-line abiraterone followed by third-line cabazitaxel (4.9 months; *p* = 0.488), though the sample size was small [19]. Our results are not directly comparable with the above studies as we did not distinguish between the specific AR-targeted therapies received and did not condition on the number of lines of therapy received when the cohorts were established, as did the cited studies. Keeping these differences in study design in mind, while we found no statistically significant differences in terms of OS for patients in the cabazitaxel versus AR-targeted therapy cohorts in the full patient population, we did find that the subgroup of patients with worse disease prognosis at initiation of second-line therapy benefitted from receiving second-line cabazitaxel versus second-line AR-targeted therapy, post-docetaxel. These findings are consistent with a separate analysis of this study, which assessed the effectiveness of second-line taxane chemotherapy versus an alternative AR-targeted therapy in chemotherapy-naïve patients who received first-line AR-targeted therapy. In this patient population, second-line chemotherapy (docetaxel or cabazitaxel), compared with second-line AR-targeted therapy, was also associated with improved OS in patients with worse prognostic features, namely low albumin or hemoglobin levels [20]. Findings from our study, in the post-docetaxel and chemotherapy-naïve settings, are in line with the results of a separate study that found an association between second-line cabazitaxel and prolonged PFS in patients who had a worse prognosis, i.e., a higher Gleason score (≥8), compared with patients who had a lower Gleason score (<7; HR: 0.36; 95% CI 0.18–0.72; *p* = 0.004) [21].

A key strength of this study is that it is one of the largest of its kind. Moreover, as it is based on the EMRs of patients treated in a USA community oncology setting, it provides a unique insight into the link between real-world treatment patterns and patient outcomes. Use of EMR data also allowed for inclusion of clinical parameters typically unavailable in other information resources such as administrative healthcare claims databases. Despite these strengths, there were a number of limitations to our methodology that should be considered. Firstly, therapy information in the EMR database was obtained from physician prescription data rather than from filled prescription files taken from claims databases. This means that prescriptions for oral medications were not necessarily filled and taken as directed. Secondly, some of the fields in the EMR database were not well populated; dummy variables indicating missing values were therefore included in the multivariate analyses (residual confounding may, however, still be present as only recorded variables could be adjusted for as a means to control for confounding). For comparison of treatment effectiveness, confounding by indication may be present and may affect the validity of the results. For instance, cabazitaxel administration may have been influenced by disease severity. All analyses were therefore adjusted for indicators of disease severity. Finally, our study focused on community oncology settings, and results may therefore not be generalizable to care received in other settings.

In conclusion, although no significant difference in OS was observed between the two treatment groups overall, our findings suggest that following treatment with docetaxel, second-line cabazitaxel versus second-line AR-targeted therapy may be associated with an improved survival benefit in patients with poorer prognostic features (Halabi high-risk score or low albumin/hemoglobin levels). Prospective randomized trials are warranted to validate these results. Future research should also consider other approved therapies, such as sipuleucel-T and radium-223, as well as features such as adverse events and health-related quality of life.
